# Immunobiology of cancer-associated fibroblasts in the context of radiotherapy

**DOI:** 10.1186/s12967-021-03112-w

**Published:** 2021-10-18

**Authors:** Turid Hellevik, Rodrigo Berzaghi, Kristin Lode, Ashraful Islam, Inigo Martinez-Zubiaurre

**Affiliations:** 1 Department of Radiation Oncology, University Hospital of Northern Norway, Tromsø, Norway; 2 Department of Clinical Medicine, Faculty of Health Sciences, UiT-the Arctic University of Norway, Tromsø, Norway

**Keywords:** Cancer-associated fibroblasts, CAFs, Immunosuppression, Ionizing radiation, Radiotherapy, Tumor microenvironment, TME

## Abstract

Radiotherapy (RT) still represents a mainstay of treatment in clinical oncology. Traditionally, the effectiveness of radiotherapy has been attributed to the killing potential of ionizing radiation (IR) over malignant cells, however, it has become clear that therapeutic efficacy of RT also involves activation of innate and adaptive anti-tumor immune responses. Therapeutic irradiation of the tumor microenvironment (TME) provokes profound cellular and biological reconfigurations which ultimately may influence immune recognition. As one of the major constituents of the TME, cancer-associated fibroblasts (CAFs) play central roles in cancer development at all stages and are recognized contributors of tumor immune evasion. While some studies argue that RT affects CAFs negatively through growth arrest and impaired motility, others claim that exposure of fibroblasts to RT promotes their conversion into a more activated phenotype. Nevertheless, despite the well-described immunoregulatory functions assigned to CAFs, little is known about the interplay between CAFs and immune cells in the context of RT. In this review, we go over current literature on the effects of radiation on CAFs and the influence that CAFs have on radiotherapy outcomes, and we summarize present knowledge on the transformed cellular crosstalk between CAFs and immune cells after radiation.

## Introduction

After more than 100 years at service, radiotherapy (RT) still represents a predominant and cost-effective treatment modality in modern cancer care [1], securing around 40% of cancer cures when used alone or in combinatory strategies [2]. Recently, immense technological advances, in particular novel concepts like stereotactic body radiotherapy (SBRT) [3], has triggered a paradigm shift in treatment strategies and associated patient outcomes, where high-precision dose-delivery and minimal exposure to normal tissue is allowing intensified treatment regimens in curative settings [4, 5].

The long-standing success of clinical radiotherapy is traditionally attributed to its capacity for inducing apoptosis in neoplastic cells. However, within the carefully delineated target-volume, the complete collection of cellular and acellular components present in the tumor microenvironment (TME) is unavoidably affected by the potent beams of ionizing radiation (IR) [6, 7]. Hence, radiotherapy is naturally also triggering parallel responses such as vascular responses, altered immunity, antigen release, inflammatory processes, transient hypoxia and fibrosis, to name a few [8, 9].

From the immunological point of view, research efforts over the last decade have provided generous knowledge on the complex interplay between radiotherapy and the immune system [10–14]. This research has led to the recognition that the therapeutic effects of radiation may depend on antitumor immune responses in addition to direct cytotoxic effects [15, 16]. Given the combined tumoricidal and immunomodulatory potential effects of RT, especially when applied in high-dose hypo-fractionated regimens [17–20], exploring the synergistic combination of immune checkpoint blockade with SBRT has gained significant attention [21, 22]. Despite encouraging advances in our understanding of the immunogenic effects of RT, more knowledge is still needed to define how RT can be maximally exploited as an immunological adjuvant.

Cancer-associated fibroblasts (CAFs) represent a heterogeneous group of stromal cells in the TME [23] that are both phenotypically and epigenetically different from normal fibroblasts [24–27]. Unlike normal tissue-resident fibroblasts, CAFs are perpetually activated [28] and exert their biological effect by modulating the extracellular matrix and by secreting soluble factors such as growth factors and cytokines [29]. The presence of CAFs in the TME is correlated with increased angiogenesis, invasion and metastases, and thus associated with worse prognosis in many cancers, including colorectal, pancreatic, esophageal cancer and head and neck squamous cell carcinoma. Although considerable efforts are currently devoted to explore if and how CAFs are contributing to therapeutic resistance [25, 30], the plasticity and heterogenic nature of CAFs is certainly hampering progression in the field [23, 31], with specific subsets [30, 32–36] reported to mediate either pro-tumorigenic/immunosuppressive [30] or anti-tumorigenic/immunogenic effects [37, 38].

The sum of inflammatory stimuli, desmoplastic reactions and the highly immunosuppressive milieu brought by secretory CAFs to the TME [39, 40], are collectively affecting both recruitment and function of innate and adaptive immune cells in tumors [41–43]. Thus, although CAFs are often disregarded in immunological settings, their strong negative influence on anti-tumor responses should not be missed out on the journey towards optimized radio-immunotherapy outcomes. In radiotherapy settings, the effects of radiation on CAFs vary among different studies, and the potential role of CAFs on tumor radio-resistance is still controversial. The scope of this review is to gather existing knowledge on effects of radiation on CAFs; to elucidate the influence CAFs may exert on radiotherapy outcomes; and to summarize present knowledge on the transformed cellular crosstalk between CAFs and immune cells after radiation.

## Immunoregulatory functions of CAFs

**Fig. 1:**
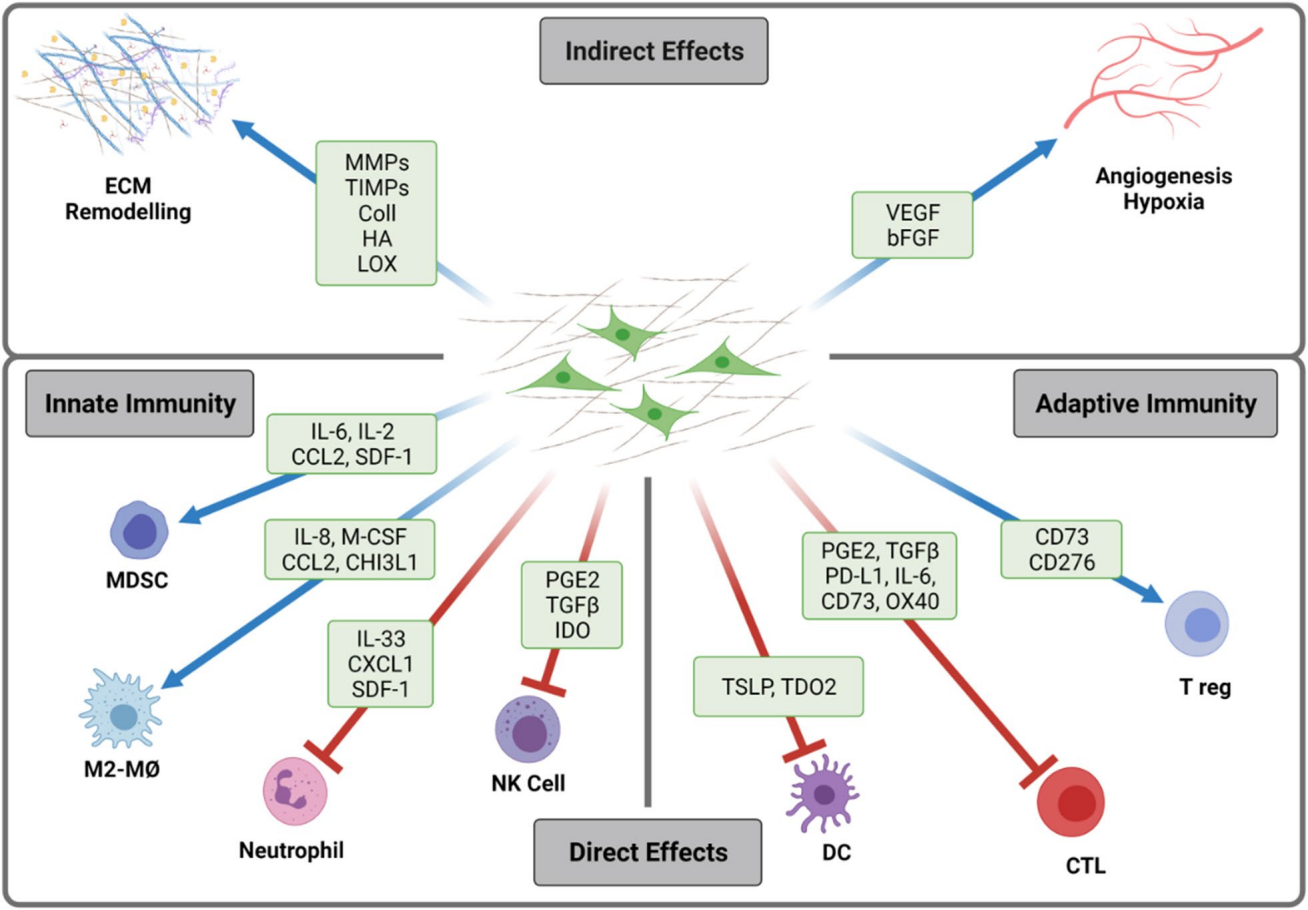
CAF-mediated immunoregulatory functions in the tumor microenvironment. Cancer-associated fibroblasts (CAFs) participate actively in the reciprocal communication with tumor and immune cells in the tumor microenvironment (TME) and are recognized contributors to immune escape by affecting recruitment and function of different innate and adaptive immune cells. Effects from CAFs on immune cells can be exerted directly via release of potent immune regulators and exosomes and/or expression of regulatory receptors on the cell surface, as well as indirectly by regulation of extracellular matrix (ECM), tissue stiffness, angiogenesis and hypoxia. Red arrows represent negative regulation, and blue arrows represent positive regulation. *bFGF* basic fibroblast growth factor; *CD73* cluster-of-differentiation-73; *CCL2* chemokine (C–C motif) ligand 2; *CHI3L1* chitinase-3 like protein 1; *coll* collagen; *CTL* cytotoxic T lymphocyte; *CXCL1* chemokine (C–X–C motif) ligand 1; *DC* dendritic cell; *ECM* extracellular matrix; *GM-CSF* granulocyte–macrophage colony-stimulating factor; *HA* hyaluronan; *IDO* Indoleamine-2,3-dioxygenase; *IL* interleukin; *LOX* lysyl oxidase; *MDSCs* myeloid-derived suppressor cells; *MMP* matrix metalloproteinases; *MØ* macrophages; *NK* cell natural killer cell; *PD-L1* programmed death ligand-1; *PGE2* prostaglandin E2; *SDF-1* stromal-derived factor-1; *TDO2* tryptophan 2,3-dioxygenase; *TGFβ* transforming growth factor beta; *T reg* regulatory T cells; *TSLP* thymic stromal lymphopoietin; *VEGF* vascular endothelial growth factor. Schematic created by BioRender

Stromal and immune cells in tumors are engaged in a bidirectional crosstalk that involve the release of soluble signal molecules as well as contact-dependent interactions (Fig. 1). Thus, on one hand, pro-inflammatory cytokines released by tumor-infiltrating immune cells, such as TNF-α and IL-1β, can favor recruitment and activation of mesenchymal progenitor cells into tumors. In turn, tumor-activated fibroblasts, i.e., CAFs, can further worsen inflammatory reactions by secreting a myriad of chemokines and cytokines that sustain chemotaxis and polarization of immune cells to the tumor bed [41, 42, 44]. Of note, resident and recruited immune cells are commonly polarized towards an immunosuppressive phenotype within the TME, that supports tumor progression and therapy resistance. Besides the direct effects exerted by CAFs on inflammatory and adaptive immune cells, CAFs participate actively in extracellular matrix (ECM) deposition, tissue stiffness and tumor angiogenesis, thus indirectly affecting migration and function of multiple immune cells subsets into the TME [45].

### Direct effects

#### Direct effects of CAFs on innate immunity

The functional interplay between CAFs and tumor-associated macrophages (TAMs) is an important determinant for tumor progression [46–48]. Stromal cells in tumors participate actively in recruitment of TAMs and their polarization towards the anti-inflammatory, tumor-supportive M2-phenotype. It has been shown that CAF-secreted soluble factors including CXCL12/SDF-1 [49], CCL2, IL-6, IL-10, IL-33 [50], granulocyte–macrophage colony-stimulating factor (GM-CSF), and chitinase-3 like protein 1 Chi3L1 [51, 52] are essential in macrophage recruitment and polarization (reviews in [44, 53]). Besides, CAFs are able to influence macrophage biology and interfere with potential therapeutic approaches to block tumor-associated macrophages (TAMs), by promoting ECM remodeling and recruiting myeloid-derived suppressor cells (MDSCs) [54]. Furthermore, CAFs can also influence neutrophil chemotaxis and polarization in the TME [55]. CAF-secreted IL-33 and CXCL12 promote recruitment of neutrophils to TME, whereas CAF-derived IL-6 induces neutrophil activation and survival through activation of IL-6/STAT3/programmed death-ligand 1 (PD-L1) signaling pathway. Increased neutrophil expression of PD-L1 culminates in impaired T cell mediated immunity. Conversely, neutrophils in tumors can mediate transformation of mesenchymal stem cells into CAFs by releasing inflammatory cytokines like IL-17, IL-23, and TNF-α [56].

Moreover, CAFs have been shown to exert direct immunoregulatory activities towards antigen-presenting dendritic cells (DCs). CAF-secreted TGF-β, tryptophan 2,3-dioxygenase (TDO2), IL-6, and thymic stromal lymphopoietin (TSLP) have been shown to directly affect DC recruitment and differentiation, promoting a tolerogenic phenotype characterized by lower expression of MHC class II molecules and co-stimulatory receptors CD40, CD80, and CD86, increased levels of suppressive cytokines and increased regulatory T cell (Treg) expansion [57–59]. These CAF-educated DCs promoted tumor infiltration of immunosuppressive Treg (CD4 + CD25 + Foxp3 +) cells and decreased production of IFN-γ from CD8 + T cells [57]. The interplay between CAFs and DCs has also been shown to affect the ability of DCs to induce differentiation of T cells into a type-2 helper T cell (Th2) phenotype in pancreatic cancer, presumably via CAF-secretion of TSLP [59].

Stromal cells and their progenitors also facilitate recruitment of MDSCs, an immature population of bone marrow-derived myeloid cells that exert potent immunosuppressive effects. CAF-secreted CXCL12/SDF-1, CCL2, IL-6, CXCL1, VEGF, TGF‐β, prostaglandin E2 (PGE2), and GM-CSF are factors thought to influence MDSC recruitment and differentiation [54, 60, 61]. In a pancreatic cancer model, CAFs could attract monocytes and further differentiate them into MDSCs via IL-6-mediated STAT3 activation [60]. Moreover, a sub-population of FAP-α expressing CAFs was shown to promote tumor growth by secretion of CCL2 and subsequent recruitment of MDSCs in murine models of hepatic cancer, liver, and lung squamous cell carcinoma [62]. Collectively, the miscellaneous effects of CAFs on the different sub-types of inflammatory cells aid in establishing an immunosuppressive environment in tumors and can polarize helper T cell responses toward an immunosuppressive Th2 profile, which further support tumor progression (Fig. 1).

#### Direct effects of CAFs on effector immune cells

CAF-mediated immunosuppression also involves inhibition of multiple effectors of anticancer immune responses. Natural killer (NK) cells play an important role in tumor immunity acting as innate effector cells [63]. CAF-secreted soluble factors including TGF-β, PGE2, Indoleamine-2,3-dioxygenase (IDO), matrix-metalloproteinases (MMPs), and surface expression of checkpoint ligands such as the poliovirus receptor (PVR/CD155) or PD-L1 may modulate NK cells immune phenotype. Several in vitro studies have document CAF-induced changes in the expression of activating receptors (NKp30, NKG2D, NKp44, NKp30, and DNAM-1), inhibitory receptors (NKG2A, KIR2DL1, and KIR3DL1), production of cytolytic granules (perforin and granzyme B) and cytokine release by NK cells [64–68].

Along similar lines, CAFs are inhibiting the activity of CD8 + cytotoxic T lymphocytes (CTL) by different means: (A) expressing immune checkpoint molecules including PD-L1 and PD-L2 and FAS ligand [69]; (B) secreting soluble mediators, e.g., CXCL12/SDF-1, IL-10, PGE2, nitric oxide and TGF-β [70], and (C) inducing metabolic rewiring through high consumption of glucose, arginine and tryptophan (all of which are required for optimal expansion of CTLs), in parallel to an elevated expression of IDO-1 [71], arginase 1 [72] and lactate [73]. In addition, CD4 + , CD25high, Foxp3 + Tregs can be recruited into tumors and expanded by CAFs either by secretion of IL-6, PGE2, and CXCL12/SDF-1 or by expressing OX40L, PD-L2, and JAM2 [33, 71]. MHC-II expressing fibroblasts have recently been described to increase Treg numbers in pancreatic cancer, through PGE2 expression and down-regulation of co-stimulatory ligands necessary to activate immune effector T cells with potential to mediate antitumor immunity [74]. In the TME, the presence of CAFs and their secretion of chemokines and cytokines such as CCL2, CCL5, CCL17, IL-1, IL-6, IL-13, and IL-26 can favor a tumor-promoting Th2 and Th17 immune response, at the expense of tumor-protective Th1 responses [75, 76].

### Indirect effects

#### CAF-mediated ECM remodeling and fibrotic reactions

Fibroblasts are engaged in connective tissue homeostasis and participate in both ECM deposition and turnover. Hence, in addition to direct cell–cell communications, CAFs may contribute to immune cell modulation indirectly by affecting interstitial pressure and tissue stiffness. In cancer settings, CAFs are main suppliers of fibrous collagens, fibronectin, elastin, laminin and ECM‐remodeling enzymes like collagen crosslinking lysyl oxidase (LOX), MMPs and tissue-inhibitors-of-metalloproteinases (TIMPs). Altogether, the wide collection of CAF-secreted molecules is affecting tissue stiffness [77] and represents a physical barrier for tumor-infiltration of blood, immune cells, drugs and (O_2_) molecules [24, 78], (review in [79]). In human pancreatic ductal adenocarcinoma (PDAC) [80] and lung cancers [81], high levels of fibrosis have been correlated with poor CD8^+^ CTL infiltration and motility [81], (review in [82]). Conversely, in a mouse model of spontaneous PDAC, deletion of collagen type-I from αSMA + CAFs induced elevated CXCL5 expression from tumor cells, immunosuppression and concomitant recruitment of MDSCs [83]. Based on a similar hypothesis, focal adhesion kinase (FAK) activity was proposed as a druggable CAF-target [84], with inhibition reducing fibrosis and rendering pancreatic cancers responsive to checkpoint immunotherapy [85]. However, similarly to collagen-I depletion [86], resistance to FAK inhibition is apparently linked to stromal depletion [87].

#### CAF-mediated effects on tumor vasculature and hypoxia

Stromal cells in tumors are also potent regulators of angiogenesis and tumor perfusion. Different mechanisms have been described for these effects including (1) secretion of pro-angiogenic factors, like VEGF, bFGF and SDF-1 [49, 88]; (2) surface expression of galectin-1 and podoplanin that upregulate VEGF in cancer cells [89, 90]; (3) activation of TGF-β signaling that triggers angiogenesis by upregulating VEGF expression on CAFs [91]; and (4) ECM remodeling [92]. Hence, indirectly, CAFs participate in the generation of hypoxic zones that in turn contribute to an immunosuppressive TME [26]. Hypoxia reportedly impair antitumor immune responses by activation of hypoxia-inducible factor-1α (HIF-1α) [93], which in turn, upregulates PD-L1 expression by MDSCs, macrophages, DCs, and tumor cells, thereby promoting T cell inactivation through the PD-1/PD-L1 axis [94, 95]. Hypoxia also triggers CD39 and CD73 ecto-nucleotidases, thereby generating extracellular (immunosuppressive) adenosine [96, 97]. Taken together, CAFs may indirectly interfere negatively with anti-tumor immunity by contributing to increased tissue stiffness and thereby promoting an immunosuppressive hypoxic microenvironment.

## Role of CAFs in radiotherapy

### Direct effects of ionizing radiation on CAF phenotype and functions

**Fig. 2:**
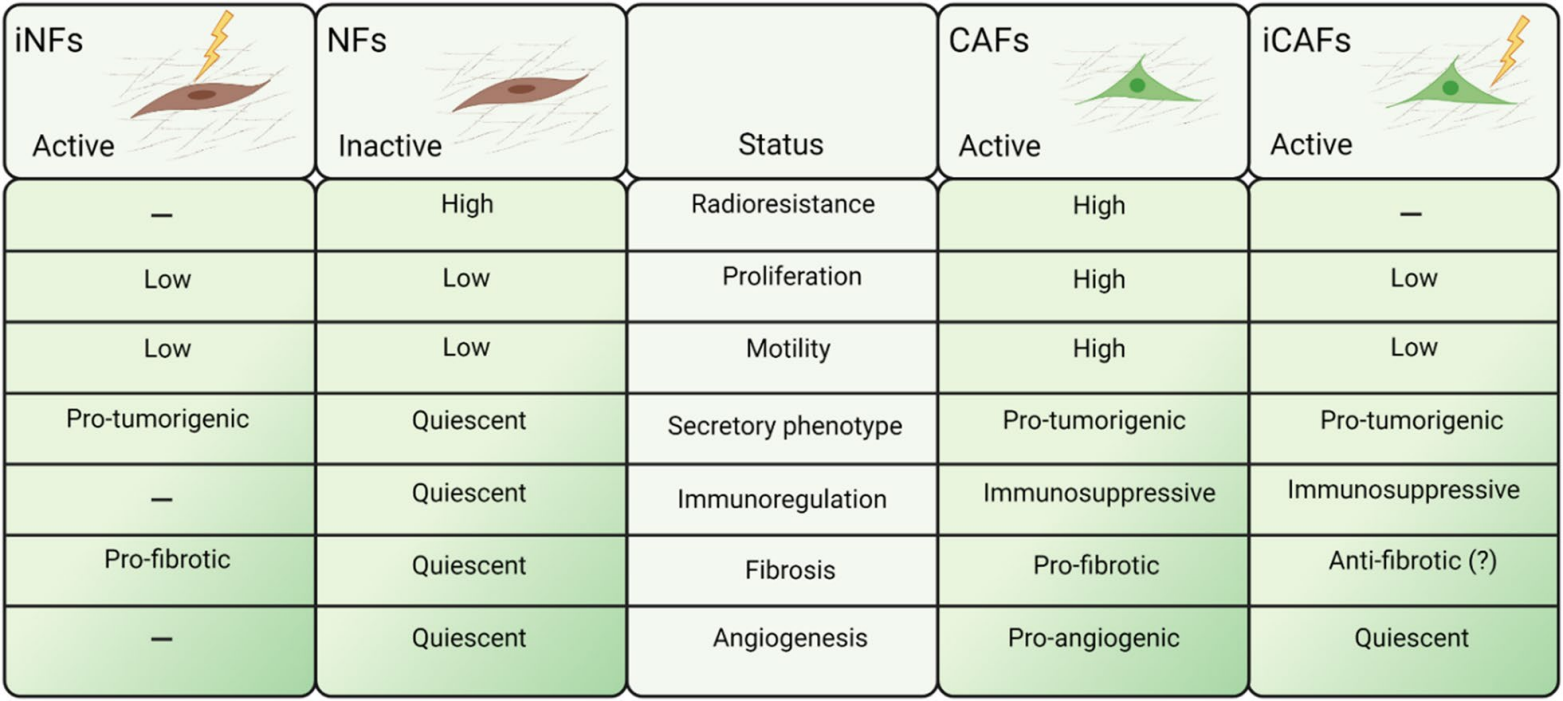
Side-by-side comparison of radiation effects exerted on normal fibroblasts and cancer-associated fibroblasts (CAFs). Compared to their normal counterparts, CAFs exhibit higher proliferation and migration rates, and actively participate in sustaining a pro-inflammatory and immunosuppressive tumor microenvironment. Exposure to ionizing radiation (IR) in vitro have been shown to activate normal tissue resident fibroblasts, rendering them more pro-tumorigenic, whereas the effects of radiation on CAF tumorigenic functions remain controversial. *NFs* normal fibroblasts; *iNFs* irradiated normal fibroblasts; *iCAFs* irradiated CAFs

CAFs in solid tumors are located intermingling and in close proximity to cancer cells, very frequently occupying areas corresponding to connective tissue bundles surrounding tumor cell nests [78]. As main elements of the supportive stromal tissue in tumors, CAFs receive the same prescribed radiation dose as tumor cells during radiotherapy. Studies investigating cytotoxic effects of IR have revealed the intrinsic radio-resistant nature of fibroblasts [98–100]. Numerous in vitro observations have confirmed that following radiation exposure, CAFs evade cell death but acquire a senescent phenotype accompanied by impaired proliferation and migration rates (Fig. 2). In the study by Tommelein et al. [101] irradiated colorectal-CAFs did not undergo cell detachment or death but demonstrated substantial DNA damage and growth delay that was maintained in long-term cultures. Non-small-cell lung carcinoma (NSCLC)-CAFs have demonstrated dose-dependent DNA damage responses following single-dose radiation (2, 6, 12 or 18 Gy), and senescence induction was proportional to the radiation dose [98]. In the same study, radiation appeared to impede CAF mobility by stabilizing focal contacts through increased surface expression of integrins [98].

Genome-wide studies conducted to map the overall changes in gene-expression undergone by irradiated fibroblasts have revealed that some critical pathways become persistently altered by radiation, including cell-cycle and proliferation, DNA-damage responses, programmed cell death, p53/p21 response genes, reactive oxygen species (ROS) scavenging, ECM remodeling and growth factors receptor signaling [102, 103].

Importantly, following radiation, CAFs maintain elevated levels of cytokine secretion but display a partially altered secretory phenotype (Fig. 2). Colorectal-CAFs have demonstrated increased secretion of numerous proteins following a fractionated low-dose regimen (10 × 1.8 Gy), including angiopoietin-like 2 (ANGPTL2) and VEGF involved in angiogenesis, along with increased levels of Dickkopf WNT signaling pathway inhibitor-1 (Dkk-1), secreted frizzled-like protein-4 (sFRP-4) and LDL receptor-related protein-6 (LRP-6) [101]. Others have found that NSCLC-CAFs respond to radiation by increased secretion of basic fibroblast growth factor (bFGF), growth arrest-specific protein 6 (GAS-6) and macrophage migratory inhibitory factor (MIF), but decreased CXCL12/SDF-1, connective tissue growth factor (CTGF) and IL-6 [104]. The secretory phenotype of irradiated CAFs seem to differ across different studies, an issue that may depend on experimental features such as tumor type, cell source or radiation regimen. In the context of cellular communication, a recent in vitro study has demonstrated that the secretion rates and the protein cargo of extracellular vesicles released by NSCLC-CAFs remains unchanged following single high dose or hypo-fractionated radiation exposure [105].

### Activation of normal fibroblasts by radiation

A frequent misconception in the field of CAFs and radiation is to generalize observations attained with CAFs and with normal (quiescent) fibroblasts. This is a highly relevant issue that deserves careful consideration. To understand the contribution of CAFs to therapeutic outcomes post-RT, the activated status of the cell pre-treatment should be considered (Fig. 2). Additionally, in contemporary radiotherapy settings, only cells residing within the delineated tumor volume or its periphery (CAFs) are exposed to the full prescribed radiation dose, whereas healthy tissue located outside the irradiated field (normal fibroblasts) may receive only residual radiation doses.

Because of the above-mentioned inaccuracies, there is a generalized view that promulgates an enhanced pro-malignant nature of irradiated fibroblasts/CAFs, irrespective of their origin. Indeed, most of the existing knowledge in this topic emerges from studies conducted with normal tissue fibroblasts or fibroblast cell-lines. The pro-malignant phenotype acquired by normal fibroblasts turning senescent post-RT has been thoroughly documented [100, 106]. Also, numerous in vitro studies have demonstrated increased invasiveness, proliferation rates and radio-resistance of tumor cells exposed to irradiated versus non-irradiated normal fibroblasts [107–109]. To these studies we must add observations on tumor bed effects when both tumorigenic and non-tumorigenic epithelial cells are transplanted into irradiated normal tissue [110, 111]. In contrast to (quiescent) normal tissue fibroblasts, tumor-reactive fibroblasts or CAFs (in non-irradiated conditions) actively produce numerous tumor-promoting molecules such as MMPs, inflammatory cytokines, pro-angiogenic factors and miscellaneous tumor-promoting growth factors [24–26] (Fig. 2). In the next chapter, we discuss radiation studies performed specifically with CAFs.

### Effects of radiotherapy on CAF pro-tumorigenic functions

In an effort to understand the role of CAFs on tumor radio-resistance, different groups have explored the direct radioprotective effects exerted by (non-irradiated) CAFs on cancer cells. Some in vitro studies have demonstrated radioprotective effects of CAF-conditioned medium on survival and colony-forming abilities of pancreatic cancer cells [112] and HeLa cells [113]. Zhang et al. [114] suggested that CAF-derived CXCL1 and the subsequent reduction in ROS scavenging enzyme superoxide dismutase-1 in cancer cells is responsible for induction of a radioresistant phenotype in esophageal squamous cancer cells (ESCC) [114]. In models of melanoma and lung cancer, elevated expression of insulin growth factor 1 (IGF-1) and chemokine CXCL12/SDF-1 by CAFs (non-irradiated) have been shown to be responsible for radioprotective effects on cancer cells [115]. Whereas in a pancreatic cancer model, authors suggest that increased expression of TFG-β and possibly other soluble factors from pancreatic stellate cells (PSCs) mediate EMT changes and acquisition of a radioresistant phenotype [116].

In addition to the general radioprotective functions assigned to CAFs, some studies claim that radiation exposure is amplifying the intrinsic radioprotective and pro-malignant effects exerted by CAFs. Upon co-culturing with irradiated CAFs, cells from ESCC were found to scatter in a dose-dependent manner, thus reflecting increased migratory behavior [117]. The effects were attributed to increased expression of hepatoma-derived growth factor (HDGF) by irradiated CAFs compared to non-irradiated controls. In another in vitro study, CAFs irradiated with single doses of 5 or 10 Gy triggered increased invasiveness of pancreatic cancer cells compared to non-irradiated CAFs [118]. Authors observed increased phosphorylation of HGF receptor in CAF-educated tumor cells, however levels of HGF in irradiated CAF-conditioned medium were unchanged [118]. Similarly, in a study by Li et al*.* [119] irradiated CAFs provoked enhanced invasive capacity of pancreatic cancer cells in co-cultures. Irradiated CAFs were found to excrete increased levels of CXCL12/SDF-1, ultimately promoting cancer cell migration, invasion and epithelial-mesenchymal transition, aiding in the overall tumor progression [119]. Again, in a pancreatic cancer model, Mantoni et al. [120] demonstrated that PSCs promote radioprotection and stimulate proliferation of pancreatic cancer cells in direct co-cultures and after co-injections in vivo. In that study, interfering with β1-integrin signaling abolished the radioprotective effects [120]. Tommelein et al. [101]found increased IGF signaling in irradiated colorectal-CAFs, and both IGF-1 and IGF-binding proteins (IGFBP2) levels were almost three-fold higher in supernatants from irradiated versus non-irradiated CAFs [101].

In contrast to the generalized view postulating a radiation-enhanced activation of CAFs, some studies document a loss of CAF pro-tumorigenic functions after irradiation. In a study by Hellevik et al. [99], the tumor enhancing effects exerted by CAFs, when co-injected with A549 lung tumor cells in xenografts, was lost when CAFs were irradiated pre-implantation [99]. Recently, Arshad et al. [121], reported that murine lung-CAFs did not modulate the intrinsic radio-sensitivity of cancer cells, and reduced TGF-β and MMPs secretion in co-culture supernatants was observed post-RT (1 × 10 Gy) [121]. In a recent study by Steer et al. [122], the radioprotective and long-term survival effects of CAFs over cancer cells were studied in 2D and 3D in vitro systems, using different sets of fibroblasts and tumor cell-lines. Results were inconsistent among different fibroblast-tumor cell combinations [122]. Similar observations were obtained after co-implantation of cells in xenografts. Authors concluded that the impact of fibroblasts on cancer cell behavior and radiation sensitivity largely depend on the respective cell type combination and that effects cannot be generalized. In clinical settings, Maaren et al*.* [123] reported that women diagnosed with early-stage breast cancer undergoing breast-conserving surgery plus radiotherapy displayed significantly improved 10 years overall and relative survival compared to women receiving mastectomy only [123]. This suggests that residual irradiated stroma is aiding in the long-term anti-tumor immunity. As a major constituent of the tumor stroma [24], CAFs and/or fibroblasts are likely to be involved in the long-term positive anti-tumor effects reported by Maaren et al*.*

## Immunoregulatory features of irradiated CAFs

**Fig. 3:**
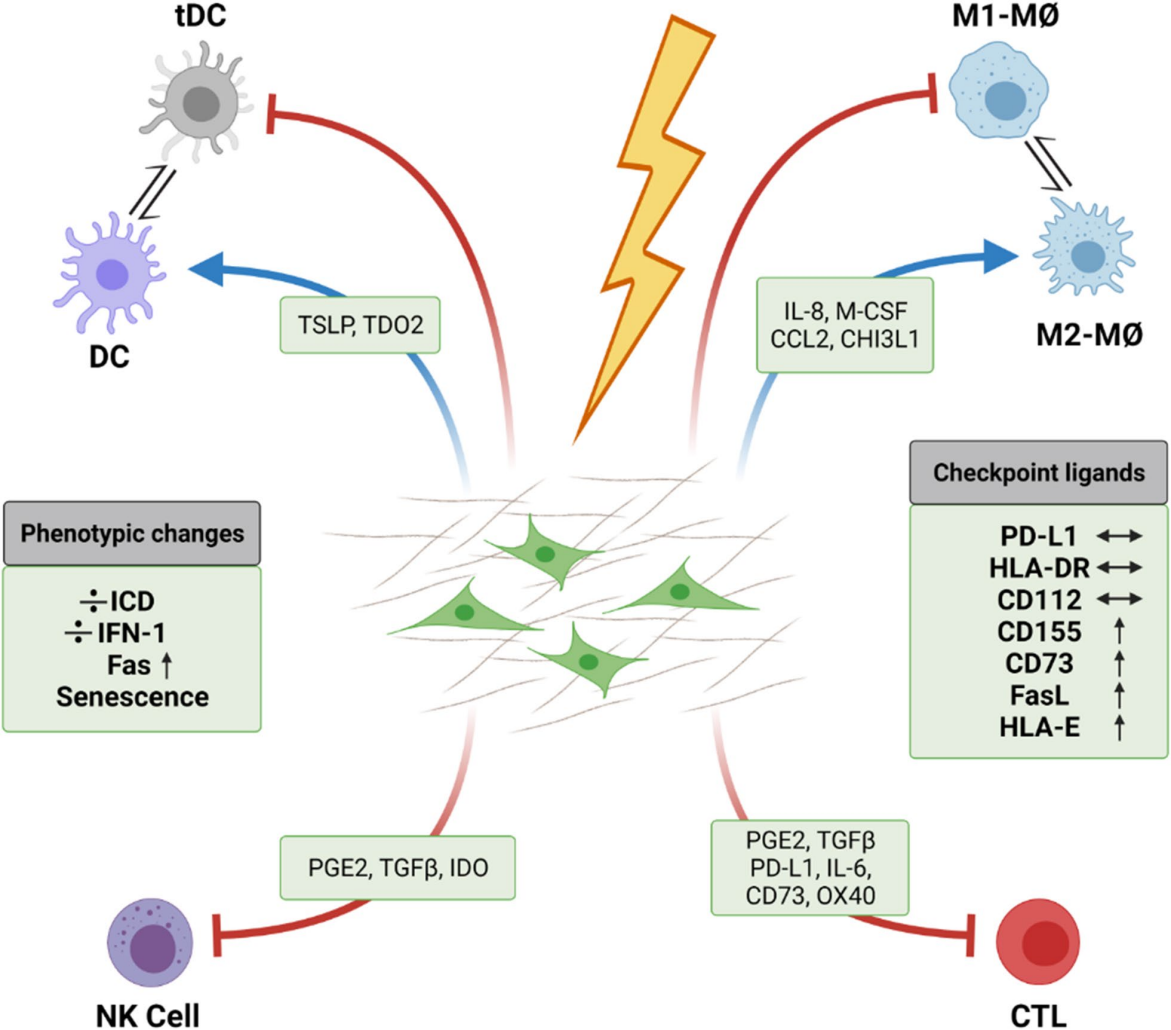
Cancer-associated fibroblasts (CAFs) maintain their immunosuppressive phenotype following exposure to ionizing radiation (IR). Radiation treatment by IR is able to induce weighty changes in the phenotype of CAFs, however recent studies have demonstrated that CAFs retain their immunosuppressive functions over different innate and adaptive immune cells after radiation treatment. The release of key immunoregulators remain constant in radiation-induced senescent CAFs. In contrast to what has been observed with tumor cells, CAFs do not undergo immunogenic cell death (ICD) and do not activate interferon type 1 (IFN-1) responses following radiation, while expression of some inhibitory surface receptors is enhanced. *CCL2* chemokine (C–C motif) ligand 2; *CHI3L1* chitinase-3 like protein 1; *CD73* cluster-of-differentiation-73; *CTL* cytotoxic T lymphocytes; *DC* dendritic cells; *FasL* Fas (or CD95) ligand; *GM-CSF* granulocyte–macrophage colony-stimulating factor; *ICD* immunogenic cell death; *IDO* Indoleamine-2,3-dioxygenase; *IFN-1* type I interferon; *IL-6* interleukin-6; *Mø* Macrophages; *M2-MØs* (anti-inflammatory) type-2 macrophages; *NK* cell natural killer cell; *PD-L1* programmed death ligand-1; *PGE2* prostaglandin E2; *TDO2* tryptophan 2,3-dioxygenase; *tDC* tolerogenic dendritic cell; *TSLP* thymic stromal lymphopoietin; *TGFb* transforming growth factor beta. Schematic created by BioRender

While the multifactorial immunoregulatory functions of CAFs have been widely studied and thoroughly documented [42, 44], CAF-mediated immunoregulation in the radiation context has been poorly investigated. As indicated earlier, CAFs are recognized as highly radioresistant, and as such, even at clinically high radiation doses, CAFs avoid cell death and instead enter into a permanent senescent state. Consequently, CAFs are not participating in anti-tumor adjuvanticity with the release of immunogenic cell death signals following RT [124]. Moreover, cultured NSCLC-CAFs do not switch on IFN type I responses after single-high dose (1 × 18 Gy) or fractionated (3 × 6 Gy) radiation [125]. However, radiation is inducing phenotypic changes in CAFs that could influence post-RT immunoregulation. Hence, IR-induced senescent NSCLC-CAFs display enhanced expression of several cell surface inhibitory ligands such as CD155, HLA-E, CD73, and Fas receptor whereas expression of other immune regulatory ligands such as PD-L1 and CD112 seem unchanged (Fig. 3) [68]. The observed phenotypic changes could affect NK cell- and T cell-mediated tumor immune attack, although differences in effector cell immune functions were not observed in (irradiated) CAFs/NK cells co-culture settings. Notably, radiation has been shown to trigger surface expression of Fas death receptor in cultured CAFs, however, this cellular response appeared insufficient to guide immune-mediated elimination of radiation-induced senescent CAFs [68].

Several studies have documented RT-mediated changes in CAF-expression of soluble immunomodulators, which could exert direct regulation on immune cell chemotaxis and/or functions. CAFs from ESCC have demonstrated enhanced expression of the chemoattractant CXCL1 post-IR [114]. Increased expression of CXCL12/SDF-1 by CAFs following irradiation has been also reported in pancreatic cancer [119], melanoma and lung cancers [115]. In the latter two studies, authors elaborate on CAF/tumor cell effects without exploring immuno-modulation, however, CXCL12/SDF-1 may exert measurable impact on immune cell recruitment and/or polarization and thus, CXCL12/SDF-1-mediated immunomodulation could potentially take place. Studies using normal fibroblasts or fibroblast cell-lines in culture have reported elevated TGF-β [107], IL-6 and IL-8 expression [106] following irradiation of cells with 1 × 12 Gy and 1 × 10 Gy, respectively. In a study by Hellevik et al. [104], a comprehensive analysis of the entire secretome of cultured NSCLC-CAFs irradiated with 1 × 18 Gy was performed using proteomics and multiplex protein assays. Results from that study demonstrated reduced SDF-1 expression and unaffected expression of miscellaneous immunomodulators including IL-6, IL-8, Il-1β, TNFα and TGF-β. Inconsistencies in outcomes across different studies could be related to variations in experimental parameters such as tumor types, cell sources, radiation doses, culturing characteristics and timing for sample collection post-IR.

As described earlier, CAF may influence immune cell infiltration and functions indirectly through ECM deposition and matrix remodeling. The fibroproliferative reactions frequently observed post-RT is the consequence of perpetuated wound healing responses [126]. Following radiation exposure, the subsequent upregulated secretion of TGF-β [127, 128] promotes activation of quiescent fibroblasts into myofibroblasts [129]. Nevertheless, participation of irradiated CAFs towards RT-induced profibrotic responses has not been thoroughly demonstrated hitherto. In an oral squamous cell carcinoma model, enhanced TGF-β expression was observed from different fibroblast cell-lines exposed to (1 × 12 Gy) [107]. However, unchanged TGF-β expression has been reported from senescent NSCLC-CAFs exposed to single-high (1 × 18 Gy) radiation dose [51, 124]. Papadopoulou et al. [100] reported enhanced expression of MMPs as partially responsible for the pro-tumorigenic effects exerted by radiation-induced senescent lung fibroblast. In contrast, Hellevik et al. [98] reported down-regulated MMP-1 from irradiated lung-CAFs, and also Arshad et al. [121] observed reduced secretion of TGF-β and MMP after simultaneous irradiation of fibroblasts and cancer cells in co-cultures (1 × 10 Gy) [121]. Accumulated evidences suggest that stress-induced senescent fibroblasts display a rather catabolic phenotype, characterized by reduced expression of structural ECM proteins and elevated expression of proteolytic enzymes [130].

### Interplay between CAFs and immune cells after radiation

The immunoregulatory functions exerted by CAFs have been extensively documented, however, as described in this review, radiation exerts substantial phenotypic changes in CAFs, so the question still remains: are the inherent immunomodulatory functions of CAFs changed after radiation exposure, and if so in which direction? Very scant knowledge exists in this respect. Four separate studies have recently been exploring how IR is affecting CAF-mediated immunoregulatory functions over different adaptive and innate immune cells in vitro (Fig. 3).

A first study, performed with white blood cells isolated from peripheral blood of randomly selected healthy donors, compared effects from control and irradiated CAFs over lymphocytes [124]. Regulation of lymphocytic activation (including both CD4 + and CD8 + T cells) was examined in proliferation, migration and cytokine release assays. In all functional assays, CAF-conditioned medium induced powerful immunosuppressive effects on activated T cells, and this effect was sustained by senescent CAFs after single-dose radiation (2 Gy or 18 Gy). Importantly, relevant immunosuppressive molecules such as PGE2, IL-6, IL-10, or TGF-β were measured in CAF-conditioned medium, but their secreted levels were unchanged post-irradiation [125].

A second study investigated how IR modulates CAF-mediated regulatory effects over exogenously polarized monocyte-derived macrophages [51]. NSCLC-CAFs were shown to inhibit pro-inflammatory features of M1-macrophages, including reduced expression of M1-surface markers, nitric oxide production, pro-inflammatory cytokines and migration rates. Radiation delivered to CAFs as single-high dose (1 × 18 Gy) or in fractioned regimens (3 × 6 Gy) did not modify their immunoregulatory features over macrophages in vitro. Of note, protein expression analyses in CAF supernatants showed that irradiated and non-irradiated CAFs secrete similar quantities of immunoregulators such as GM-CSF, monocyte chemoattractant protein-1 (MCP-1), VEGF-A, IL-4, -6, -8, -10 and Chi3L1 [51].

In a third study, CAF-mediated immunoregulatory effects on NK cells were compared with or without radiation [68]. Results revealed that cytokine-activated NK cells in direct contact with NSCLC-CAFs display a phenotype characteristic of tolerogenic NK cells, as indicated by reduced cytotoxic capacity, reduced degranulation, reduced expression of activating receptors (NKG2D, NKp46, DNAM-1) but enhanced surface expression of inhibitory receptors like NKG2A. Notably, radiation exposure to CAFs did neither improve nor worsen the overall CAF-induced immunosuppression on NK cells. In line with observations from functional assays, the release of relevant soluble immunoregulators such as PGE2, TGF-β, or IDO by control and irradiated CAFs remained unchanged. Interestingly, authors demonstrated enhanced surface expression of Fas (death receptor) and HLA-E in irradiated CAFs, however, these cellular responses turned out insufficient to initiate immune recognition and elimination by NK cells [68].

In a fourth study, effects of IR towards CAF-mediated regulation of DCs was explored [125]. Results showed that CAFs, both by conditioned medium and in co-cultures, interfere with monocyte differentiation into DCs and induce a tolerogenic phenotype on mature DCs. This was evidenced by decreased expression of classic activation markers (CD80, CD86, CD40 and HLA-DR) and reduced functional properties (migration, antigen uptake, and CD4 + T cell priming). Interestingly, IR applied in fractionated medium-doses (3 × 6 Gy) abrogated some of the CAF-mediated effects on DCs, however CAF-derived TSLP and tryptophan 2,3-dioxygenase (TDO2) levels were unchanged, suggesting that radiation-induced effects were not related to modulation of previously highlighted soluble mediators. Together, this study suggests that certain radiation regimens may modify favorably the inherent immunosuppressive functions of CAFs towards DCs [125]. The rationale behind these observations is still unknown, and the results presented in this study should also be confirmed in more complex in vivo models.

## Concluding remarks

Besides triggering direct cytotoxic effects on malignant cells, clinical radiotherapy is causing profound cellular and molecular reconfigurations in the TME that ultimately may impact tumor immune recognition [6, 7]. Numerous in vitro and in vivo studies have documented the profound changes provoked by radiation in tumor constituents such as the vasculature, desmoplasia, mesenchymal cells, inflammatory cells and bone marrow-derived progenitor cells [6, 7]. Effects of radiotherapy on CAFs vary among different studies, and the potential role of CAFs on tumor radioresistance is still controversial. Discrepancies between studies may arise due to different experimental parameters including tumor model, cell sources, in vitro culture conditions, radiation regimens and time post-treatment for data collection. In this context, generalization of results obtained with normal tissue fibroblasts, fibroblast cell-lines and CAFs isolated from tumor specimens is a frequent and non-redundant matter. To understand the contribution of CAFs to therapeutic outcomes post-RT, the activated status of the cells pre-treatment should always be considered. In addition, a considerable number of studies have explored the radioprotective effects of CAFs or their conditioned medium in non-irradiated conditions. Considering that CAFs are building blocks of the tumor mass, and therefore receive the prescribed radiation dose in full, studies exploring CAF-mediated radioprotection should consider doing it with irradiated cells.

We are gradually uncovering the important effects that radiotherapy exert on fibroblasts, and start to decipher the complex interplay between different TME elements during and after RT. However, there are clear limitations in the studies published in this field hitherto. Most of the presented studies have used single radiation doses or regimens and have collected data at specific time points, normally few hours/days post-radiation. These approaches overlook potential differences related to different RT-regimens, and disregard potential long-term effects of RT on CAFs. Besides, studies performed on in vivo models commonly use co-injections of human tumor cells and fibroblasts/CAFs in subcutaneous pockets or orthotopically in immunodeficient mice. However, in such models, human fibroblasts are rapidly replaced by host fibroblasts [99], and immunological effects are not taken into account. Transgenic mouse models, where endogenous CAFs can be regulated, could represent attractive models to explore CAF-mediated influence on RT-outcomes.

There exist a plethora of studies demonstrating that mesenchymal cells in tumors can exert powerful immunoregulation, directly affecting the polarization of immune cells within the TME and influencing their spatial localization and functionality. However, CAFs represent a highly diverse population of cells, thus while the immunosuppressive effects from CAFs are overrepresented in the literature, some studies have demonstrated specific immune-mediated tumoricidal effects orchestrated by specific CAF sub-populations [37, 38]. Future studies on the immunoregulatory roles of CAFs should embrace the heterotypic view of CAFs, identifying CAF subtypes by specific set of markers, and assigning effects to specific CAF subclasses.

In the context of radiotherapy concretely, CAF-mediated immunoregulation has been poorly investigated. Numerous in vitro studies have shown enhanced expression of immunoregulatory cytokines and growth factors by irradiated fibroblasts; however, the scenario is different when the cells in focus are CAFs instead of quiescent fibroblasts or cell lines. Another relevant finding is the upregulation of checkpoint ligands and other inhibitory receptors on irradiated CAFs, a circumstance that could play a role in contact-dependent effector immune cells regulation. Furthermore, recent studies based on (irradiated) CAF/immune cell co-cultures have shown comparable immunoregulatory effects from irradiated and control CAFs over different types of immune cells, accompanied by similar expression of soluble immunomodulators. Collectively, these in vitro studies propose equivalent immunoregulatory abilities by irradiated and non-irradiated CAFs. Considering CAFs as one of the most notorious immunosuppressive elements in the TME and that radiation does not revert or may even enhance CAF-mediated immunosuppressive functions, strategies to specifically target CAF subtypes or CAF-derived factors should be considered to gain the full potential of RT as an immune adjuvant. In recent years, a wide range of CAF-targeting strategies have been tested in preclinical and clinical settings [25]. The approaches are very diverse and comprise agents targeting ECM components (e.g., PEGPH20/hyaluronidase and FG-309 mAb/anti-CTGF), CAF-specific contenders (anti-FAP antibodies, FAP vaccines or CAR T cells), pathway-specific inhibitors (TGFBR inhibitors, CXCR4 inhibitors or NF-kB inhibitors) and drugs that reprogram CAFs to a quiescent state (e.g., calcipotriol, tranilast). Recent advances on CAF molecular and functional heterogeneity open new avenues for cancer treatment. Novel strategies are now designed to target CAF-secreted tumor promoting or immunosuppressive molecules (IL-6, TGFb, etc.…) or inhibiting subtype specific signaling that would ablate a particular CAF population. In summary, recent observations from pre-clinical models suggest that targeting CAF could increase immunotherapy efficacy. However, it remains unclear which features of the heterogeneous CAF phenotype are more important to their role in immune evasion in human tumors.

## Data Availability

Not applicable.

## References

[1] Chargari C (2016). Optimize and refine therapeutic index in radiation therapy: overview of a century. Cancer Treat Rev.

[2] Sharma RA (2016). Clinical development of new drug-radiotherapy combinations. Nat Rev Clin Oncol.

[3] Finazzi T, Schneiders FL, Senan S (2021). Developments in radiation techniques for thoracic malignancies. Eur Respir Rev.

[4] Desai NB, Laine AM, Timmerman RD (2017). Stereotactic ablative body radiotherapy (SAbR) for oligometastatic cancer. Br J Radiol.

[5] Griffin RJ (2020). Understanding high-dose, ultra-high dose rate, and spatially fractionated radiation therapy. Int J Radiat Oncol Biol Phys.

[6] Hellevik T, Martinez-Zubiaurre I (2014). Radiotherapy and the tumor stroma: the importance of dose and fractionation. Front Oncol.

[7] Barker HE (2015). The tumour microenvironment after radiotherapy: mechanisms of resistance and recurrence. Nat Rev Cancer.

[8] Martinez-Zubiaurre I, Hellevik T (2018). Transformed immunosuppressive networks of the irradiated tumor stroma. Front Immunol.

[9] Monjazeb AM (2020). Effects of radiation on the tumor microenvironment. Semin Radiat Oncol.

[10] Vanpouille-Box C, Formenti SC, Demaria S (2018). Toward precision radiotherapy for use with immune checkpoint blockers. Clin Cancer Res.

[11] Arina A, Gutiontov SI, Weichselbaum RR (2020). Radiotherapy and immunotherapy for cancer: from “systemic” to “multisite”. Clin Cancer Res.

[12] De Martino M, Daviaud C, Vanpouille-Box C (2021). Radiotherapy: an immune response modifier for immuno-oncology. Semin Immunol.

[13] Jagodinsky JC, Harari PM, Morris ZS (2020). The promise of combining radiation therapy with immunotherapy. Int J Radiat Oncol Biol Phys.

[14] Schaue D (2017). A century of radiation therapy and adaptive immunity. Front Immunol.

[15] Rodriguez-Ruiz ME (2020). Immunological impact of cell death signaling driven by radiation on the tumor microenvironment. Nat Immunol.

[16] Ngwa W (2018). Using immunotherapy to boost the abscopal effect. Nat Rev Cancer.

[17] Frey B (2017). Hypofractionated irradiation has immune stimulatory potential and induces a timely restricted infiltration of immune cells in colon cancer tumors. Front Immunol.

[18] Vanpouille-Box C (2017). DNA exonuclease Trex1 regulates radiotherapy-induced tumour immunogenicity. Nat Commun.

[19] Vanpouille-Box C, Formenti SC, Demaria S (2017). TREX1 dictates the immune fate of irradiated cancer cells. Oncoimmunology.

[20] Dewan MZ (2009). Fractionated but not single-dose radiotherapy induces an immune-mediated abscopal effect when combined with anti-CTLA-4 antibody. Clin Cancer Res.

[21] Bernstein MB (2016). Immunotherapy and stereotactic ablative radiotherapy (ISABR): a curative approach?. Nat Rev Clin Oncol.

[22] Gutiontov SI (2020). Cytoreduction and the optimization of immune checkpoint inhibition with radiation therapy. Int J Radiat Oncol Biol Phys.

[23] Cremasco V (2018). FAP delineates heterogeneous and functionally divergent stromal cells in immune-excluded breast tumors. Cancer Immunol Res.

[24] Kalluri R (2016). The biology and function of fibroblasts in cancer. Nat Rev Cancer.

[25] Chen X, Song E (2019). Turning foes to friends: targeting cancer-associated fibroblasts. Nat Rev Drug Discov.

[26] Sahai E (2020). A framework for advancing our understanding of cancer-associated fibroblasts. Nat Rev Cancer.

[27] Bhowmick NA, Neilson EG, Moses HL (2004). Stromal fibroblasts in cancer initiation and progression. Nature.

[28] Erez N (2010). Cancer-associated fibroblasts are activated in incipient neoplasia to orchestrate tumor-promoting inflammation in an NF-kappaB-dependent manner. Cancer Cell.

[29] Liu T (2019). Cancer-associated fibroblasts build and secure the tumor microenvironment. Front Cell Dev Biol.

[30] Su S (2018). CD10(+)GPR77(+) cancer-associated fibroblasts promote cancer formation and chemoresistance by sustaining cancer stemness. Cell.

[31] Helms E, Onate MK, Sherman MH (2020). Fibroblast heterogeneity in the pancreatic tumor microenvironment. Cancer Discov.

[32] Ohlund D (2017). Distinct populations of inflammatory fibroblasts and myofibroblasts in pancreatic cancer. J Exp Med.

[33] Costa A (2018). Fibroblast heterogeneity and immunosuppressive environment in human breast cancer. Cancer Cell.

[34] Huelsken J, Hanahan D (2018). A subset of cancer-associated fibroblasts determines therapy resistance. Cell.

[35] Pereira BA (2019). CAF subpopulations: a new reservoir of stromal targets in pancreatic cancer. Trends Cancer.

[36] Bartoschek M (2018). Spatially and functionally distinct subclasses of breast cancer-associated fibroblasts revealed by single cell RNA sequencing. Nat Commun.

[37] Ozdemir BC (2014). Depletion of carcinoma-associated fibroblasts and fibrosis induces immunosuppression and accelerates pancreas cancer with reduced survival. Cancer Cell.

[38] McAndrews KM (2021). alphaSMA(+) fibroblasts suppress Lgr5(+) cancer stem cells and restrain colorectal cancer progression. Oncogene.

[39] Davidson S (2021). Fibroblasts as immune regulators in infection, inflammation and cancer. Nat Rev Immunol.

[40] Kraman M (2010). Suppression of antitumor immunity by stromal cells expressing fibroblast activation protein-alpha. Science.

[41] Servais C, Erez N (2013). From sentinel cells to inflammatory culprits: cancer-associated fibroblasts in tumour-related inflammation. J Pathol.

[42] Barrett R, Pure E (2020). Cancer-associated fibroblasts: key determinants of tumor immunity and immunotherapy. Curr Opin Immunol.

[43] Turley SJ, Cremasco V, Astarita JL (2015). Immunological hallmarks of stromal cells in the tumour microenvironment. Nat Rev Immunol.

[44] Monteran L, Erez N (2019). The dark side of fibroblasts: cancer-associated fibroblasts as mediators of immunosuppression in the tumor microenvironment. Front Immunol.

[45] Elahi-Gedwillo KY (2019). Antifibrotic therapy disrupts stromal barriers and modulates the immune landscape in pancreatic ductal adenocarcinoma. Cancer Res.

[46] Comito G (2014). Cancer-associated fibroblasts and M2-polarized macrophages synergize during prostate carcinoma progression. Oncogene.

[47] Takahashi H (2017). Cancer-associated fibroblasts promote an immunosuppressive microenvironment through the induction and accumulation of protumoral macrophages. Oncotarget.

[48] Gok Yavuz B (2019). Cancer associated fibroblasts sculpt tumour microenvironment by recruiting monocytes and inducing immunosuppressive PD-1(+) TAMs. Sci Rep.

[49] Orimo A (2005). Stromal fibroblasts present in invasive human breast carcinomas promote tumor growth and angiogenesis through elevated SDF-1/CXCL12 secretion. Cell.

[50] Shani O (2020). Fibroblast-derived IL33 facilitates breast cancer metastasis by modifying the immune microenvironment and driving type 2 immunity. Cancer Res.

[51] Berzaghi R (2019). Fibroblast-mediated immunoregulation of macrophage function is maintained after irradiation. Cancers.

[52] Cohen N (2017). Fibroblasts drive an immunosuppressive and growth-promoting microenvironment in breast cancer via secretion of Chitinase 3-like 1. Oncogene.

[53] Ziani L, Chouaib S, Thiery J (2018). Alteration of the antitumor immune response by cancer-associated fibroblasts. Front Immunol.

[54] Kumar V (2017). Cancer-associated fibroblasts neutralize the anti-tumor effect of CSF1 receptor blockade by inducing PMN-MDSC infiltration of tumors. Cancer Cell.

[55] Cheng Y (2018). Cancer-associated fibroblasts induce PDL1+ neutrophils through the IL6-STAT3 pathway that foster immune suppression in hepatocellular carcinoma. Cell Death Dis.

[56] Zhang J (2020). Tumor-educated neutrophils activate mesenchymal stem cells to promote gastric cancer growth and metastasis. Front Cell Dev Biol.

[57] Cheng JT (2016). Hepatic carcinoma-associated fibroblasts induce IDO-producing regulatory dendritic cells through IL-6-mediated STAT3 activation. Oncogenesis.

[58] Hsu YL (2016). Lung cancer-derived galectin-1 contributes to cancer associated fibroblast-mediated cancer progression and immune suppression through TDO2/kynurenine axis. Oncotarget.

[59] De Monte L (2011). Intratumor T helper type 2 cell infiltrate correlates with cancer-associated fibroblast thymic stromal lymphopoietin production and reduced survival in pancreatic cancer. J Exp Med.

[60] Mace TA (2013). Pancreatic cancer-associated stellate cells promote differentiation of myeloid-derived suppressor cells in a STAT3-dependent manner. Cancer Res.

[61] Deng Y (2017). Hepatic carcinoma-associated fibroblasts enhance immune suppression by facilitating the generation of myeloid-derived suppressor cells. Oncogene.

[62] Yang X (2016). FAP promotes immunosuppression by cancer-associated fibroblasts in the tumor microenvironment via STAT3-CCL2 signaling. Cancer Res.

[63] Huntington ND, Cursons J, Rautela J (2020). The cancer-natural killer cell immunity cycle. Nat Rev Cancer.

[64] Li T (2012). Hepatocellular carcinoma-associated fibroblasts trigger NK cell dysfunction via PGE2 and IDO. Cancer Lett.

[65] Li T (2013). Colorectal carcinoma-derived fibroblasts modulate natural killer cell phenotype and antitumor cytotoxicity. Med Oncol.

[66] Balsamo M (2009). Melanoma-associated fibroblasts modulate NK cell phenotype and antitumor cytotoxicity. Proc Natl Acad Sci USA.

[67] Ziani L (2017). Melanoma-associated fibroblasts decrease tumor cell susceptibility to NK cell-mediated killing through matrix-metalloproteinases secretion. Oncotarget.

[68] Yang N (2020). Irradiated tumor fibroblasts avoid immune recognition and retain immunosuppressive functions over natural killer cells. Front Immunol.

[69] Lakins MA (2018). Cancer-associated fibroblasts induce antigen-specific deletion of CD8(+) T Cells to protect tumour cells. Nat Commun.

[70] Feig C (2013). Targeting CXCL12 from FAP-expressing carcinoma-associated fibroblasts synergizes with anti-PD-L1 immunotherapy in pancreatic cancer. Proc Natl Acad Sci USA.

[71] Curran TA (2014). IDO expressing fibroblasts promote the expansion of antigen specific regulatory T cells. Immunobiology.

[72] Ino Y (2013). Arginase II expressed in cancer-associated fibroblasts indicates tissue hypoxia and predicts poor outcome in patients with pancreatic cancer. PLoS ONE.

[73] Sun K (2019). Oxidized ATM-mediated glycolysis enhancement in breast cancer-associated fibroblasts contributes to tumor invasion through lactate as metabolic coupling. EBioMedicine.

[74] Elyada E (2019). Cross-species single-cell analysis of pancreatic ductal adenocarcinoma reveals antigen-presenting cancer-associated fibroblasts. Cancer Discov.

[75] Liao D (2009). Cancer associated fibroblasts promote tumor growth and metastasis by modulating the tumor immune microenvironment in a 4T1 murine breast cancer model. PLoS ONE.

[76] Barnas JL (2010). Reciprocal functional modulation of the activation of T lymphocytes and fibroblasts derived from human solid tumors. J Immunol.

[77] Pankova D (2016). Cancer-associated fibroblasts induce a collagen cross-link switch in tumor stroma. Mol Cancer Res.

[78] LeBleu VS, Kalluri R (2018). A peek into cancer-associated fibroblasts: origins, functions and translational impact. Dis Model Mech.

[79] Nia HT, Munn LL, Jain RK (2020). Physical traits of cancer. Science.

[80] Watt J, Kocher HM (2013). The desmoplastic stroma of pancreatic cancer is a barrier to immune cell infiltration. Oncoimmunology.

[81] Salmon H (2012). Matrix architecture defines the preferential localization and migration of T cells into the stroma of human lung tumors. J Clin Invest.

[82] Freeman P, Mielgo A (2020). Cancer-associated fibroblast mediated inhibition of CD8+ cytotoxic t cell accumulation in tumours: mechanisms and therapeutic opportunities. Cancers.

[83] Chen Y (2021). Type I collagen deletion in alphaSMA(+) myofibroblasts augments immune suppression and accelerates progression of pancreatic cancer. Cancer Cell.

[84] Zaghdoudi S (2020). FAK activity in cancer-associated fibroblasts is a prognostic marker and a druggable key metastatic player in pancreatic cancer. EMBO Mol Med.

[85] Jiang H (2016). Targeting focal adhesion kinase renders pancreatic cancers responsive to checkpoint immunotherapy. Nat Med.

[86] Chen Y (2021). Type I collagen deletion in alphaSMA(+) myofibroblasts augments immune suppression and accelerates progression of pancreatic cancer. Cancer Cell.

[87] Jiang H (2020). Development of resistance to FAK inhibition in pancreatic cancer is linked to stromal depletion. Gut.

[88] Hayashi Y (2016). p53 functional deficiency in human colon cancer cells promotes fibroblast-mediated angiogenesis and tumor growth. Carcinogenesis.

[89] Bektas S (2010). CD24 and galectin-1 expressions in gastric adenocarcinoma and clinicopathologic significance. Pathol Oncol Res.

[90] Schoppmann SF (2013). Podoplanin expressing cancer associated fibroblasts are associated with unfavourable prognosis in adenocarcinoma of the esophagus. Clin Exp Metastasis.

[91] Yang J (2016). Vascular mimicry formation is promoted by paracrine TGF-beta and SDF1 of cancer-associated fibroblasts and inhibited by miR-101 in hepatocellular carcinoma. Cancer Lett.

[92] Lederle W (2010). MMP13 as a stromal mediator in controlling persistent angiogenesis in skin carcinoma. Carcinogenesis.

[93] Noman MZ (2015). Hypoxia: a key player in antitumor immune response. A review in the theme: cellular responses to hypoxia. Am J Physiol Cell Physiol.

[94] Noman MZ (2014). PD-L1 is a novel direct target of HIF-1alpha, and its blockade under hypoxia enhanced MDSC-mediated T cell activation. J Exp Med.

[95] Barsoum IB (2014). A mechanism of hypoxia-mediated escape from adaptive immunity in cancer cells. Cancer Res.

[96] Allard B (2017). The ectonucleotidases CD39 and CD73: novel checkpoint inhibitor targets. Immunol Rev.

[97] Allard B (2020). The adenosine pathway in immuno-oncology. Nat Rev Clin Oncol.

[98] Hellevik T (2012). Cancer-associated fibroblasts from human NSCLC survive ablative doses of radiation but their invasive capacity is reduced. Radiat Oncol.

[99] Grinde MT (2017). Ionizing radiation abrogates the pro-tumorigenic capacity of cancer-associated fibroblasts co-implanted in xenografts. Sci Rep.

[100] Papadopoulou A, Kletsas D (2011). Human lung fibroblasts prematurely senescent after exposure to ionizing radiation enhance the growth of malignant lung epithelial cells in vitro and in vivo. Int J Oncol.

[101] Tommelein J (2018). Radiotherapy-activated cancer-associated fibroblasts promote tumor progression through paracrine IGF1R activation. Cancer Res.

[102] Rodningen OK (2005). Microarray analysis of the transcriptional response to single or multiple doses of ionizing radiation in human subcutaneous fibroblasts. Radiother Oncol.

[103] Martinez-Zubiaurre I (2013). Tumorigenic responses of cancer-associated stromal fibrioblasts after ablative radiotherapy: a transcriptome-profiling study. J Cancer Ther.

[104] Hellevik T (2013). Changes in the secretory profile of NSCLC-associated fibroblasts after ablative radiotherapy: potential impact on angiogenesis and tumor growth. Transl Oncol.

[105] Berzaghi R (2021). Secretion rates and protein composition of extracellular vesicles released by cancer-associated fibroblasts after radiation. J Radiat Res.

[106] Rodier F (2009). Persistent DNA damage signalling triggers senescence-associated inflammatory cytokine secretion. Nat Cell Biol.

[107] Kamochi N (2008). Irradiated fibroblast-induced bystander effects on invasive growth of squamous cell carcinoma under cancer-stromal cell interaction. Cancer Sci.

[108] Tsai KK (2009). Low-dose radiation-induced senescent stromal fibroblasts render nearby breast cancer cells radioresistant. Radiat Res.

[109] Patel ZS (2012). Ionizing radiation enhances esophageal epithelial cell migration and invasion through a paracrine mechanism involving stromal-derived hepatocyte growth factor. Radiat Res.

[110] Tsai KK (2005). Cellular mechanisms for low-dose ionizing radiation-induced perturbation of the breast tissue microenvironment. Cancer Res.

[111] Barcellos-Hoff MH, Ravani SA (2000). Irradiated mammary gland stroma promotes the expression of tumorigenic potential by unirradiated epithelial cells. Cancer Res.

[112] Hwang RF (2008). Cancer-associated stromal fibroblasts promote pancreatic tumor progression. Cancer Res.

[113] Chu TY (2014). Crosstalk with cancer-associated fibroblasts increases the growth and radiation survival of cervical cancer cells. Radiat Res.

[114] Zhang H (2017). CAF-secreted CXCL1 conferred radioresistance by regulating DNA damage response in a ROS-dependent manner in esophageal squamous cell carcinoma. Cell Death Dis.

[115] Wang Y (2017). Cancer-associated fibroblasts promote irradiated cancer cell recovery through autophagy. EBioMedicine.

[116] Al-Assar O (2014). Contextual regulation of pancreatic cancer stem cell phenotype and radioresistance by pancreatic stellate cells. Radiother Oncol.

[117] Bao CH (2015). Irradiated fibroblasts promote epithelial-mesenchymal transition and HDGF expression of esophageal squamous cell carcinoma. Biochem Biophys Res Commun.

[118] Ohuchida K (2004). Radiation to stromal fibroblasts increases invasiveness of pancreatic cancer cells through tumor-stromal interactions. Cancer Res.

[119] Li D (2016). Radiation promotes epithelial-to-mesenchymal transition and invasion of pancreatic cancer cell by activating carcinoma-associated fibroblasts. Am J Cancer Res.

[120] Mantoni TS (2011). Pancreatic stellate cells radioprotect pancreatic cancer cells through beta1-integrin signaling. Cancer Res.

[121] Arshad A, Deutsch E, Vozenin MC (2015). Simultaneous irradiation of fibroblasts and carcinoma cells repress the secretion of soluble factors able to stimulate carcinoma cell migration. PLoS ONE.

[122] Steer A (2019). Impact of cancer-associated fibroblast on the radiation-response of solid xenograft tumors. Front Mol Biosci.

[123] van Maaren MC (2016). 10 year survival after breast-conserving surgery plus radiotherapy compared with mastectomy in early breast cancer in the Netherlands: a population-based study. Lancet Oncol.

[124] Gorchs L (2015). Cancer-associated fibroblasts from lung tumors maintain their immunosuppressive abilities after high-dose irradiation. Front Oncol.

[125] Berzaghi R (2021). Ionizing radiation curtails immunosuppressive effects from cancer-associated fibroblasts on dendritic cells. Front Immunol.

[126] Rubin P (1995). A perpetual cascade of cytokines postirradiation leads to pulmonary fibrosis. Int J Radiat Oncol Biol Phys.

[127] Finkelstein JN (1994). Early alterations in extracellular matrix and transforming growth factor beta gene expression in mouse lung indicative of late radiation fibrosis. Int J Radiat Oncol Biol Phys.

[128] Rube CE (2000). Dose-dependent induction of transforming growth factor beta (TGF-beta) in the lung tissue of fibrosis-prone mice after thoracic irradiation. Int J Radiat Oncol Biol Phys.

[129] Straub JM (2015). Radiation-induced fibrosis: mechanisms and implications for therapy. J Cancer Res Clin Oncol.

[130] Mavrogonatou E (2019). Extracellular matrix alterations in senescent cells and their significance in tissue homeostasis. Matrix Biol.

